# Association of interleukin-17A and chemokine/vascular endothelial growth factor-induced angiogenesis in newly diagnosed patients with bladder cancer

**DOI:** 10.1186/s12865-024-00612-4

**Published:** 2024-03-21

**Authors:** Ali Moadab, Mohammad Rafie Valizadeh, Alireza Nazari, Hossein Khorramdelazad

**Affiliations:** 1 Department of Immunology, School of Medicine, Rafsanjan University of Medical Sciences, Rafsanjan, Iran; 2 Non-Communicable Diseases Research Center, Rafsanjan University of Medical Sciences, Rafsanjan, Iran; 3 Department of Surgery, School of Medicine, Rafsanjan University of Medical Sciences, Rafsanjan, Iran

**Keywords:** IL-17A, CXCR2, VEGF, TGF-β, Bladder cancer

## Abstract

**Background:**

The human interleukin-17 (IL-17) family comprises IL-17A to IL-17 F; their receptors are IL-17RA to IL-17RE. Evidence revealed that these cytokines can have a tumor-supportive or anti-tumor impact on human malignancies. The purpose of this study was to assess the expression of *CXCR2*, *IL-17RA*, and *IL-17RC* genes at the mRNA level as well as tissue and serum levels of IL-17A, vascular endothelial growth factor (VEGF), and transforming growth factor β (TGF-β) in patients with bladder cancer (BC) compared to control.

**Results:**

This study showed that gene expression of *IL-17RA*, *IL-17RC*, and *CXCR2* in the tumoral tissue of BC patients was significantly upregulated compared with normal tissue. The findings disclosed a significant difference in the serum and tissue concentrations of IL-17A, VEGF, and TGF-β between the patient and the control groups, as well as tumor and normal tissues.

**Conclusion:**

This study reveals notable dysregulation of *CXCR2, IL-17RA*, and *IL-17RC* genes, alongside changes in IL-17A, VEGF, and TGF-β levels in patients with BC than in controls. These findings indicate their possible involvement in BC development and their potential as diagnostic and therapeutic targets.

## Introduction

Bladder cancer (BC) is known as one of the most common types of urinary tract cancer, affecting 40,000 people worldwide every year with a very high mortality rate [1]. Several factors, including smoking habits, workplace exposure, age, gender, and genetics, can contribute to the BC pathophysiology [2]. On the other hand, immune system components and mediators such as immune cells, cytokines, growth factors, and their subsequent responses can correspondingly lead to tumor eradication or progression [3, 4]. Cytokines and chemokines are small glycoproteins involved in numerous immune and non-immune cell biological phenomena [5, 6]. These immune mediators can also cause cell growth, proliferation, survival, differentiation, migration, and apoptosis or necrosis [4, 7, 8]. Moreover, cytokines can participate in anti-tumoral responses considering the status of the tumor microenvironment (TME); however, in some pathologic states such as chronic inflammation, depending on the balance and concentrations of pro-and anti-inflammatory mediators, the activation state of surrounding cells and expression of their receptors, cytokines can be effective in tumor progression [9–12]. Studies have revealed that among these cytokines, the IL-17 family (IL-17A to IL-17 F) and their receptor (IL-17RA to IL-17RE) are involved in Th17 responses, and they can participate in pro-tumor or anti-tumor responses depending on their phenotype [13].

In addition, it is well known that IL-17A and IL-17 F can bind to IL-17RA and IL-17RC receptors, and these types of ligands and receptors can be involved in cytokine release, neutrophil recruitment, inflammation, and vascular endothelial growth factor (VEGF)-associated angiogenesis [14, 15]. Furthermore, IL‑17 A may induce chemokine‑induced angiogenesis (CXCL8/CXCR2 axis) and promote tumor progression independent of the VEGF pathway [16]. As a tumor escape mechanism, tumor cells release high levels of transforming growth factor β (TGF-β) and CXCL8 to enhance growth and invasion via inducing angiogenesis [17]. Induction of VEGF by IL-17A stimulates TGF-β production and angiogenesis [18]. Accordingly, this study aimed to explore the role of the IL-17A/IL-17RA/C axis in VEGF- and CXCR2-mediated angiogenesis in patients with BC.

## Materials and methods

The study was designed as a cross-sectional study and enrolled forty-five male patients with confirmed BC referred to Moradi Hospital affiliated with Rafsanjan University of Medical Sciences, Rafsanjan, Iran, from January 2022 to November 2022. All the patients had invasive BC (T2-T4) regarding the pathological findings. Moreover, forty-two age and gender-matched healthy subjects without a history of urological disorders were enrolled in this study. According to the potential impression of chronic inflammation and taking anti-inflammatory medicines on the outcomes of the study, healthy subjects with severe infections in the last six months, an acute or chronic inflammatory disorder, allergies, asthma, rheumatoid arthritis (RA), liver cirrhosis, trauma, Crohn’s disease, diabetes, ulcerative colitis, pituitary tumors, multiple sclerosis, urinary tract infections, other malignant neoplasia, subjects who might have been exposed to industrial chemicals of aromatic amines such as benzidine and beta-naphthylamine were excluded. Additionally, patients with BC who had received the Bacillus Calmette-Guerin (BCG) vaccine or mitomycin were excluded from the study. Tissue and blood samples were taken from all newly diagnosed patients before taking chemotherapy or other anti-cancer treatments.

Following physical examination, urological examination, ultrasonography (US) of the bladder, intravenous urography (IVU), and cystoscopy, a definite indication for surgery (transurethral resection-TUR) was given. BC tumoral tissue and adjacent normal tissue samples (bladder epithelial tissues at a distance of over five cm from the edge of tumoral tissues) were obtained [19]. The grade and stage of BC were determined based on the TNM (Tumor Nodules Metastases) classification of malignant tumors and a histopathological examination [20]. All patients had a high grade of BC following histopathological examination, based on cancer progression potential. The Ethics Committee of Rafsanjan University of Medical Sciences approved the protocol of this study. The informed consent was explained to each participant, and the oral and written consent forms were obtained from patients before the surgery and sample collection.

### Quantitative RT-PCR

According to the manufacturer’s instructions, total RNA content was extracted from the obtained normal and tumoral tissues using an RNA extraction kit (Cat.# 9767, Takara, Kyoto, Japan). Extracted total RNA integrity and purity were also measured via agarose gel electrophoresis and UV spectrophotometry (260/280 and 260/230 ratios). The cDNA was generated from the yielded total RNA employing PrimeScript™ II 1st strand cDNA Synthesis Kit (TaKaRa Bio, Shiga, Japan). The RT-PCR was subsequently performed in a 20 µL solution containing 10 µL SYBR® Premix Ex Taq TM (Takara, Japan), 0.4 µL ROX, 3 µL normalized cDNA, and 1 µM of forward and reverse primers, specific for *IL-17RA*, *IL-17RC*, *CXCR2*, and *Actin-β* as well as 4.6 µL nuclease-free water. The primer sequences are shown in Table 1. Each RT-PCR assay was also completed in triplicate using a thermal cycler ABI Step One Plus® system (Applied Biosystems™, USA). Additionally, the relative *IL-17RA*, *IL-17RC*, and *CXCR2* levels were calculated by 2^−∆∆Ct^ [4]. Finally, RT-PCR length products were confirmed via agarose gel electrophoresis and a 50 to 500 base pairs (bp) DNA ladder marker (Jena Bioscience, Germany).

**Table 1:** The sequences of primers used in the study

Gene	Forward	Reverse	product length
*Actin β*	TCACCATGGATGATGATATCGC	ATAGGAATCCTTCTGACCCATGC	164 bp
*IL-17RA*	CATCACGGGCATCTCCATCC	AGGCAGGCCATCGGTGTAT	127 bp
*IL-17RC*	GTTCAGGTGAACAGCTCGGA	GTACAGCCACTGGGTTCCAA	149 bp
*CXCR2*	TGGATTTCCCCCTTGCAACC	AAATCCTGACTGGGTCGCTG	200 bp

bp: base per

### Protein extraction from tumor tissues

After tissue isolation, tumor and normal tissue samples were ground in ice-cold RIPA buffer using a tissue homogenizer to disrupt cell membranes and release proteins. The homogenate was then centrifuged at 12,000 rpm for 15 min at 4 °C to pellet cell debris and collect the supernatant containing soluble proteins. Protein concentration in the supernatant was determined using a Bradford assay kit (Thermo Fisher, 87,792, USA) according to the manufacturer’s instructions. The protein extract was aliquoted into appropriate volumes and stored at -80 °C until further analysis.

### Cytokine assay

To measure the serum levels of IL-17A, the peripheral blood (3 mL) was collected from the patients and healthy subjects before the surgery. The serum was separated by centrifugation and then stored at -80^º^C for cytokine assay. Tumor and normal tissues were isolated during the surgery, and tissue preparation was performed according to the ELISA kit manufacturer’s instructions. Serum and tissue concentrations of IL-17A were detected using the ELISA kit (Cusabio, CSB-E12819h, USA). The kits’ sensitivity was 15.6 pg/mL, with a 62.5 pg/ml − 4000 pg/ml assay range. Tissue levels of TGF-β were also measured using the ELISA kit (Invitrogen, BMS249-4, USA). The kit’s sensitivity was 8.6 pg/mL with a 31.25–2000 pg/mL assay range. Additionally, the tissue level of VEGF was also detected using the ELISA kit (Invitrogen, EHVEGFACL; USA), and the sensitivity of the kit was reported to be less than 10 pg/mL with an 8.23–6000 pg/mL assay range. All procedures were done according to the manufacturer’s instructions. Inter- and intra-assays produced scores were CV < 15% and CV < 5%, respectively.

### Statistical analysis

Statistical analysis was conducted using GraphPad Prism version 9 software (GraphPad Software, San Diego, CA, USA) to assess differences between groups and evaluate diagnostic accuracy. The Kolmogorov-Smirnov test assessed data normality. The parametric unpaired T-test compared groups for normally distributed data, while the non-parametric Mann-Whitney U test was used for non-normally distributed data. Receiver Operating Characteristic (ROC) curve analysis, employing the Wilson/Brown method with a 95% confidence interval, evaluated the diagnostic performance of variables, calculating the area under the ROC curve (AUC) and determining sensitivity, specificity, positive predictive value (PPV), and negative predictive value (NPV) at various cutoff points. Optimal cutoff values were identified using the Youden index or maximizing sensitivity and specificity. Data were presented as mean ± standard deviation (SD), and statistical significance was set at *p* < 0.05 for all tests.

## Results

All the subjects (*n* = 87) were matched in age and gender. A total of 45 patients with BC (mean age of 62.15 ± 11.37 years) and 42 healthy subjects (mean age of 58.55 ± 12.31 years) were enrolled in this study. The mean age in the control group was lower than BC patients, but this difference was not statistically significant (*P* = 0.32) (Table 2).

**Table 2:** Some demographic data in BC patients and control group

Variable	Control	BC patients	P value
*n*	42	45	-
Age (years, M ± SD)	58.55 ± 12.31	62.15 ± 11.37	NS
Smoking	10 (33.3%)	23 (76.6%)	0.01*
Tumor size (cm)	-	2.9 ± 0.8	-
Drinking	3 (10%)	5 (16.6%)	ns
Tumor grades (low/high)	0/0	0/30	-
Pesticide handling (%)	0 (0)	32 (71.1%)	-
Duration exposure to pesticides (years, M ± SD)	-	10.2 ± 2.4	-

ns: not significant, *significant differences (P ˂ 0.05)

**Fig. 1:**
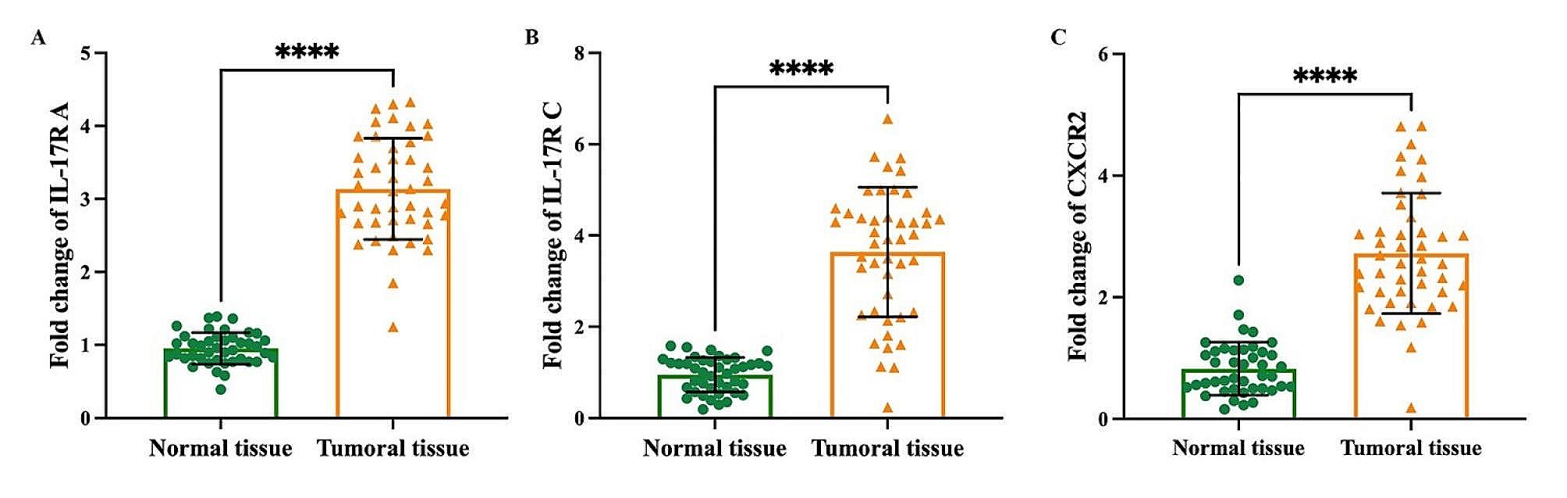
Demonstrates alteration in mRNA level of *IL-17RA* (**A**) *IL-17RC* (**B**), and *CXCR2* (**C**) in normal and tumoral tissues of patients with BC. All experiments were performed in triplicate. Data are presented as mean ± SD. ****significant differences (P ˂ 0.0001)

**Fig. 2:**
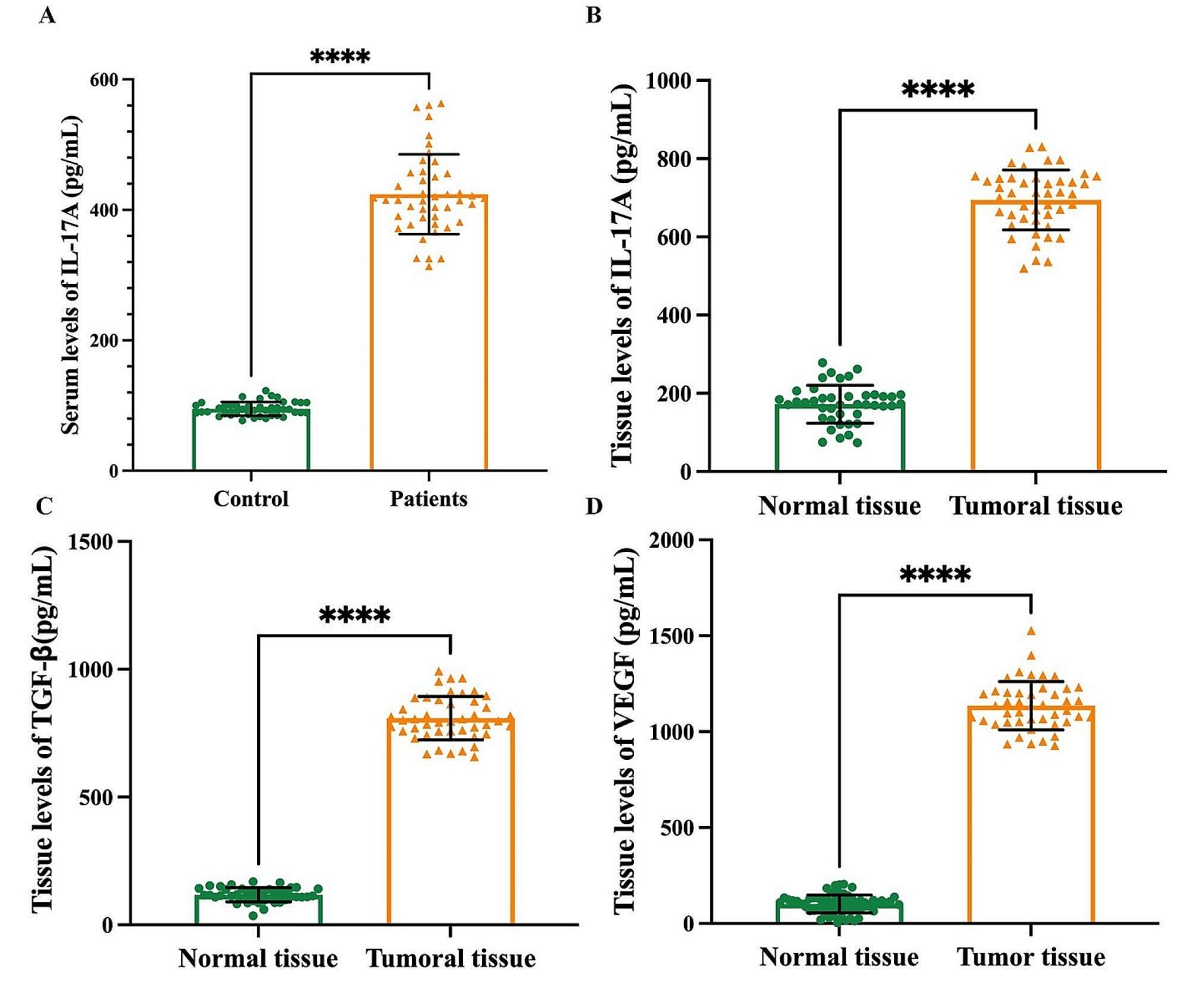
demonstrates serum levels of IL-17A (**A**), and tissue levels of IL-17 A (**B**), TGF-β (**C**), and VEGF (**D**) in patients with BC compared to control. Results are presented as mean ± SD. ****Statistically significant difference between patients with BC and other groups (P ˂ 0.0001)

The data obtained from the RT-PCR showed a significant upregulation in the expression of *IL-17RA* (Fig. 1A), *IL-17RC* (Fig. 1B), and *CXCR2* (Fig. 1C), in the tumoral tissue of BC patients compared to normal tissue (*p* < 0.0001). Moreover, this study revealed a significant increase in the serum levels of IL-17A in BC patients (12.87 ± 3.26 pg/mL) compared with the control group (95.06 ± 10.55 pg/mL) (*p* < 0.0001). The difference between the control and BC patients was 328.9 ± 9.582 pg/mL (Fig. 2A). In addition, tissue levels of IL-17A were remarkably higher in BC patients (694.5 ± 76.67 pg/mL) than in normal tissues (172.3 ± 48.28 pg/mL) with a 522.2 ± 13.85 pg/mL difference between the normal and tumoral tissues (*p* < 0.0001) (Fig. 2B). Measuring the tissue concentrations of VEGF showed that there was a significant increase in the tissue levels of VEGF in patients with BC (1136 ± 126.2 pg/mL) in comparison with the normal control tissues (99.04 ± 49.27 pg/mL) (*P* < 000.1) (Fig. 2C). The difference between the mean of tumor and normal tissues in the VEGF test was 1037 ± 20.82 pg/mL. In this study, the tissue levels of TGF-β were measured in normal and tumor tissues of patients with BC. The outcomes revealed a significant elevation in the tissue level of TGF-β in BC patients (808.7 ± 84.64 pg/mL) compared to the normal control tissue (117.6 ± 27.52 pg/mL) with a 691.0 ± 13.69 pg/mL difference between the normal and tumoral tissues (*p* < 0.0001) (Fig. 2D).

**Fig. 3:**
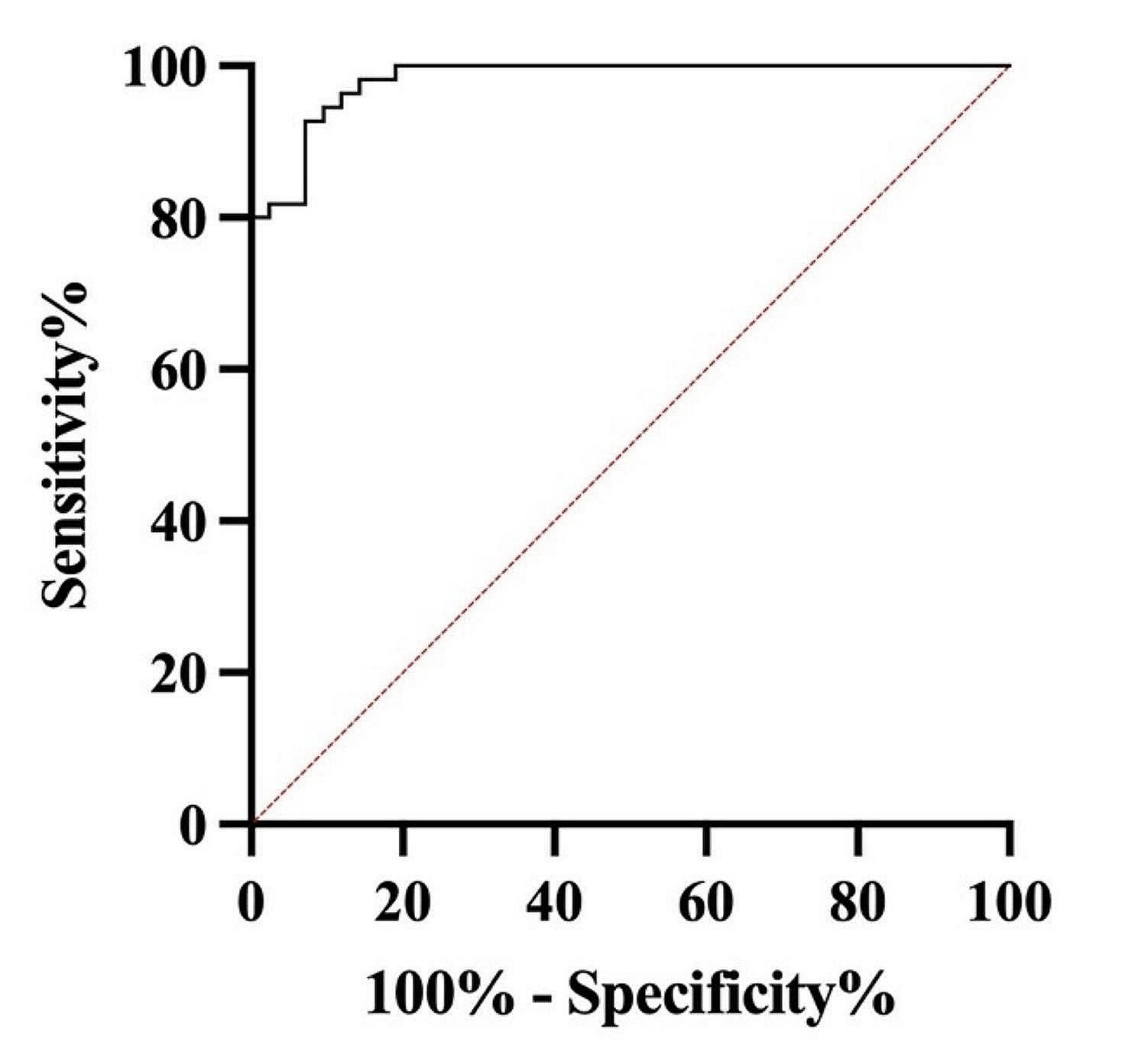
The ROC curve analysis demonstrates the diagnostic efficacy of a cutoff value of 123.6 pg/mL in distinguishing between controls (*n* = 42) and patients (*n* = 45). The area under the ROC curve is 0.9818 (95% CI: 0.9627 to 1.000, *p* < 0.0001), indicating outstanding discriminatory power. Notably, no data were missing for either controls or patients, ensuring the reliability of the analysis. These results suggest that the proposed cutoff value offers high sensitivity and specificity, underscoring its utility as a dependable tool for differentiating the two groups in this study

The receiver operating characteristic (ROC) curve analysis revealed a promising diagnostic performance for the cutoff value of 123.6 pg/mL, with an impressive area under the ROC curve of 0.9818 (95% CI: 0.9627 to 1.000, *p* < 0.0001), indicating excellent discrimination between controls (*n* = 42) and patients (*n* = 45). There were no missing data for either controls or patients, ensuring the robustness of the analysis. These findings suggest that the proposed cutoff value exhibits high sensitivity and specificity, making it a reliable tool for distinguishing between the two groups in this study (Fig. 3).

## Discussion

There is no direct effect of IL-17A on endothelial cell proliferation, but it can make fibroblasts and tumor cells produce proangiogenic factors [21]. As a stimulator of VEGFA production in tumors, IL-17A has been identified as angiogenesis-promoting in several types of cancer, including non-small-cell lung cancer and colorectal cancer [22–24]. C-X-C chemokines, by activating the CXCR2, in addition to VEGFA, have been implicated in facilitating tumor angiogenesis. As a 7-transmembrane G protein-coupled receptor (7GPCR), CXCR2 is responsible for chemotaxis in immune responses and is upregulated in inflammatory conditions, such as RA, psoriasis, and atherosclerosis [25]. CXCR2 is expressed on microvascular endothelial cells, in addition to a subset of leukocytes, and it has been implicated in endothelial cell chemotaxis and angiogenesis. ELR^+^ CXC chemokines, such as CXCL1/2/3/5/6/8, are known to be proangiogenic [26, 27]. Gastric cancers have been shown to have higher levels of CXCL8, while renal cell carcinomas have been shown to have higher levels of CXCL1/3/5/8, which enhances angiogenesis and tumorigenesis [28]. Additionally, CXCL8, released by activated stellate cells, has been linked to hepatocellular carcinoma (HCC) angiogenesis [29]. A significant finding is that IL-17A can influence tumor growth and angiogenesis in non-small cell lung cancer despite not affecting VEGFA production [30]. This study showed that the gene expression of *IL-17RA, IL-17RC*, and *CXCR2* in tumor tissues was significantly higher than in normal tissues isolated from patients with BC. In parallel with these findings, an investigation reported that IL-17RA and IL-17RC expression were considerably upregulated in patients with BC [31]. Correspondingly, there was a remarkable increase in the expression of IL-17A, VEGF, and TGF-β in the serum and tissue of BC patients compared to the control. Considering that IL-17RA and IL-17RC are specific receptors for IL-17A as well as angiogenic properties of IL-17A, it seems that an increase in the expression of these types of receptors may contribute to tumor progression through VEGF- and CXCR2-dependent angiogenesis along with chronic inflammatory consequences [32].

The infiltration of immune cells in response to cytokines and chemokines secreted from the tumor site in BC patients can also induce tumor-supportive or anti-tumor immune responses, depending on the TME signals and condition [33]. Studies have also shown that inflammation could be considered a double sword and have a crucial role in tumor progression and regression [34]. Differentiation of CD4^+^ Th0 to Th17 also leads to the production of IL-17 [35]; however, there are contradictory data in this regard [36]. Previous studies have reported that infiltrated macrophages, endothelial cells, fibroblasts, and even tumor cells in the TME can be the main sources of IL-17A and IL-17 F [37]. Our results showed that the expression of *CXCR2* was significantly upregulated in tumor tissues than in normal tissues. This finding indicates that IL-17A can induce angiogenesis in a VEGF-independent manner [30]. IL-17A can selectively elevate the expression of chemokines with angiogenic properties by epithelial cells as well as tumor cells, such as CXCL1, CXCL5, CXCL6, and CXCL8 [30, 38]. Recently, it has been shown that following the ligation of these chemokines, such as CXCL1 to CXCR2, tumor-associated immune cells can recruit into the tumor milieu to suppress anti-tumor immune responses and also activate the angiogenic processes [39]. Furthermore, the CXCL1/CXCR2 axis can induce PMN-myeloid-derived suppressor cells (MDSCs) accumulation in the TME and induce CD8^+^ T cell exhaustion, which may offer a potential therapeutic strategy for cancer therapy [40].

IL-17A-induced VEGF plays a pivotal role in fibroblast-mediated angiogenesis [41]. In line with these facts, this study showed that the tumoral tissue levels of VEGF were increased significantly compared with normal tissue in BC patients. Therefore, it can be guessed that the activation of angiogenic pathways by IL-17A cannot be dependent only on CXC chemokines or VEGF [42, 43].

The IL-17A and VEGF loop can also induce TGF-β expression by immunosuppressive and tumor cells, stimulating VEGF receptors on endothelial cells, angiogenesis, tumor growth, and metastasis [44–46]. Some studies have shown that VEGF, in addition to its angiogenic role, can inhibit the function of T cells and the activation of dendritic cells (DCs) in the TME [47]. In the present study, the tissue level of VEGF increased significantly compared to normal tissue. In parallel with our results, a study reported that high levels of TGF-β are implicated in the proliferation and invasiveness of tumor cells in BC patients [48]. As mentioned, this type of angiogenesis that initiates with IL-17A might also stimulate anti-tumor responses by creating a channel through new vessels to infiltrate immune cells and apply their anti-tumor activity into solid tumor tissues [15]. IL-17A has been shown to stimulate the expression and release of inflammatory cytokines and chemokines by neutrophils and macrophages [49]. In line with our results, it has been reported that the circulatory levels of inflammatory cytokines such as IL-6, IL-17A, and IL-23 were remarkably increased in BC patients. Furthermore, the mRNA expression of IL-17A and retinoic acid-related orphan receptor gamma t (RORγt) was significantly upregulated in PBMCs and tissues of patients with BC [50].

Moreover, in this study, the ROC curve analysis revealed a strong diagnostic performance for the specified cutoff value, indicating excellent discrimination between control and patient groups. These findings emphasize the cutoff value’s high sensitivity and specificity, rendering it a dependable tool for distinguishing between the two groups. However, further studies with bigger sample sizes are required to confirm the possibility of using serum levels of IL-17A as a biomarker for patients with BC [46].

Finally, it should be noted that one of the strengths of this study is enrolling newly diagnosed BC patients without the use of drugs or other cancer therapeutic approaches such as radiotherapy. However, there were limitations in this study, such as a lack of access to more patients, as well as additional diagnostic methods, such as immunohistochemistry or western blot, to evaluate the protein levels of the studied variables.

## Conclusion

In conclusion, the present study investigated the expression levels of *IL-17RA*, *IL-17RC*, *CXCR2*, IL-17A, VEGF, and TGF-β in BC patients compared to healthy controls. Significant upregulation was observed in the tumoral tissue of BC patients for *IL-17RA, IL-17RC, CXCR2*, IL-17A, VEGF, and TGF-β. Moreover, serum and tissue levels of IL-17A, as well as VEGF and TGF-β tissue levels, were significantly elevated in BC patients compared to controls. These findings indicate the studied mediators could be involved in the pathogenesis of BC. Importantly, the ROC curve analysis demonstrated the promising diagnostic performance of IL-17A, indicating excellent discrimination between BC patients and controls. It suggests that the proposed cutoff value holds high sensitivity and specificity, making it a possible tool for distinguishing between the two groups in this study. However, further investigations with larger sample sizes are needed to confirm the possibility of using serum levels of IL-17A as a diagnostic biomarker for patients with BC. These findings suggest the potential of the investigated mediators as diagnostic and therapeutic targets.

## Data Availability

The datasets generated and/or analyzed during the current study are not publicly available due to personal privacy but are available from the corresponding author upon reasonable request.

## Abbreviations

IL-17: Interleukin-17
VEGF: Vascular endothelial growth factor
TGF-β: Transforming growth factorβ
BC: Bladder cancer
TME: Tumor microenvironment
RA: Rheumatoid arthritis
BCG: Bacillus Calmette-Guerin
US: Ultrasonography
IVU: Intravenous urography
7GPCR: 7-transmembrane G protein-coupled receptor
HCC: Hepatocellular carcinoma
DC: dendritic cell
RORγt: Retinoic acid-related orphan receptor gamma t
MDSC: Myeloid-derived suppressor cell
